# Dataset of chemical elements concentrations in snow samples collected in Jelgava City (Latvia) in 2021, 2022, 2023

**DOI:** 10.1016/j.dib.2025.112197

**Published:** 2025-10-17

**Authors:** Jovita Pilecka-Ulcugaceva, Anda Bakute, Maris Bertins, Arturs Viksna, Sindija Frienberga, Kristaps Siltumens, Inga Grinfelde

**Affiliations:** a Latvia University of Life Sciences and Technologies, Liela Street 2, Jelgava, LV-3001, Latvia; b University of Latvia, Jelgavas Street 1, Riga, LV-1004, Latvia; c Lietuvos Inžinerijos Kolegija | Higher Education Institution, Tvirtovės al. 35, Kaunas, LT-50155, Lithuania

**Keywords:** Urban air quality, Air pollution, Atmospheric deposition, Contaminants

## Abstract

This article presents datasets on the concentrations of chemical elements in snow samples collected in the city of Jelgava. Snow samples were collected on January 9, 2021, February 4, 2022, and January 9, 2023. Snow was allowed to accumulate for at least six days before sampling.

Snow samples were collected at 59 monitoring points in Jelgava and at one site outside the city, which served as a control. The collected snow samples were analysed using ICP-MS. The following elements were analysed in the samples: aluminum (Al), silicon (Si), chromium (Cr), manganese (Mn), iron (Fe), nickel (Ni), copper (Cu), zinc (Zn), arsenic (As), molybdenum (Mo), cadmium (Cd), barium (Ba), tungsten (W), and lead (Pb). All datasets from the 2021–2023 sampling campaigns are openly available in the Mendeley Data repository.

The concentrations showed high variability, with Zn ranging from 3.44 to 35.12 µg/L (median 7.5 µg/L) and Pb up to 3.77 µg/L in 2021, while the control site showed a Pb value of 0.95 µg/L, reflecting differences between urban sites and the rural reference point.

The compiled dataset has fundamental scientific value, as it provides a reliable basis for local and regional analyses of urban air quality. In addition, it supports the evaluation of the city’s green infrastructure and its role in improving air quality. The dataset is also valuable for assessing the effectiveness of pollution mitigation measures implemented in Jelgava and for identifying priority areas where additional actions may be needed.

Specifications Table

**Table:** 

Subject	Earth & Environmental Sciences
Specific subject area	The urban air quality
Type of data	Table, Raw, Analysed
Data collection	Snow samples were collected in clean polyethylene containers, melted at room temperature, and analysed for chemical elements using an Agilent 8900 ICP-QQQ with Micro-mist nebulizer and He collision/reaction cell. Calibration used multi-element standards traceable to NIST SRM with blank correction. Internal standards ensured measurement stability.
Data source location	Institution: Latvia University of Life Sciences and Technologies City: Jelgava Country: Latvia The latitude and longitude of the collected sample points are listed in the data repository (Mendeley Data).
Data accessibility	**Please note:** A total of three years of data are submitted - 2021, 2022, 2023: Repository name: **2021 snow data in the city of Jelgava (Mendeley Data)** Data identification number: 10.17632/2y6vwhw389.2 Direct URL to data: https://data.mendeley.com/datasets/2y6vwhw389/2 Repository name: **2022 snow data in the city of Jelgava (Mendeley Data)** Data identification number: 10.17632/9k6pwfycxh.1 Direct URL to data: https://data.mendeley.com/datasets/9k6pwfycxh/1 Repository name: **2023 snow data in the city of Jelgava (Mendeley Data)** Data identification number: 10.17632/y8445nryvr.4 Direct URL to data: https://data.mendeley.com/datasets/y8445nryvr/4
Related research article	None.

## 1 Value of the Data

• These data are important for understanding the spatial distribution of chemical elements in Jelgava and other Latvian cities, helping to identify pollution sources affecting air, water, and soil.
• They are also relevant for public health, as urban air pollution is linked to health risks. Knowing where pollutants accumulate supports targeted risk reduction and infrastructure planning.
• For urban development, the data highlight pollution hotspots and trends, informing sustainable transport and green infrastructure decisions.
• In scientific research, the data support both local and European studies on urban pollution and its effects, enabling interdisciplinary collaboration.
• Thanks to regular collection, the dataset allows for long-term monitoring and policy assessment, helping to evaluate pollution control measures in line with EU environmental goals.
• Finally, the data can be used for air quality modelling, improving simulations of pollutant dispersion and supporting initiatives by the EEA and Copernicus programme.

## 2 Background

The original motivation for compiling this dataset was to monitor the presence and spatial distribution of trace elements in snow within the urban environment of Jelgava, Latvia. The research builds on the theoretical framework of atmospheric deposition studies, where snow is used as a passive sampler to capture airborne pollutants, particularly in winter conditions when other sampling methods may be limited. The methodological background is based on standardized environmental sampling techniques for snow, followed by acidification and elemental analysis using Inductively Coupled Plasma Mass Spectrometry (ICP-MS). The dataset was generated to support ongoing efforts to understand pollutant accumulation patterns in urban areas and to provide high-resolution environmental data for further research.

This data article complements the associated research article by offering access to the complete dataset of chemical element concentrations, along with detailed information on sample locations and preparation methods. It adds value by enabling other researchers to replicate the methodology, compare results across regions, or integrate the dataset into larger-scale environmental assessments and modelling efforts.

## 3 Data Description

This article describes a dataset obtained from a field survey of snow samples collected in and around Jelgava city during the winter seasons of 2021 (Data identification number: 10.17632/2y6vwhw389.1) [1], 2022 (Data identification number: 10.17632/9k6pwfycxh.1) [2] and 2023 (Data identification number: 10.17632/y8445nryvr.1) [3]. Raw data on the concentrations of chemical elements (Al, Si, Cr, Mn, Fe, Ni, Cu, Zn, As, Mo, Cd, Ba, W, Pb) in snow samples from these seasons are provided on the Mendeley data website. Concentrations are expressed in micrograms per liter (µg/L). An Excel table is available on the website, where the first column indicates the monitoring point number, and the second and third columns indicate the geographical coordinates (WGS84) of each location. The fourth column indicates the snow sample number of each monitoring point, and the following columns indicate the measured concentrations of individual chemical elements. Data are available for 59 urban monitoring sites in Jelgava city and one rural control site in Mežciems (sample site 60) for each sampling year (Table 1, Table 2).

**Table 1:** Summary of trace element concentrations (µg/L) in snow samples from Jelgava City, Latvia, including minimum, maximum, median, and mean values for 2021–2023*.*

Trace element	2021				2022				2023			
	Minimum	Maximum	Median	Mean	Minimum	Maximum	Median	Mean	Minimum	Maximum	Median	Mean
**Al**	4.34	345.85	12.28	32.58	1.22	1911.65	21.82	64.43	2.54	176.80	7.83	17.31
**Si**	0.20	347.79	11.63	38.37	10.64	2793.58	66.13	156.03	0.01	217.30	10.79	24.11
**Cr**	0.03	1.39	0.05	0.13	0.05	4.17	0.05	0.16	0.05	5.75	0.07	0.74
**Mn**	1.59	48.64	5.06	8.45	0.80	161.37	5.08	10.09	0.68	22.28	3.69	4.64
**Fe**	10.11	621.98	43.38	87.10	0.29	548.43	5.07	16.72	1.34	399.91	13.15	33.79
**Ni**	0.08	14.21	0.15	0.51	0.07	5.02	0.21	0.33	0.03	2.82	0.13	0.32
**Cu**	0.01	18.16	0.80	1.58	0.25	20.25	0.98	1.59	0.00	14.20	0.76	1.53
**Zn**	3.44	35.12	7.50	10.02	0.21	13.46	0.77	1.28	0.96	44.96	5.47	7.71
**As**	0.16	1.36	0.23	0.26	0.01	0.13	0.01	0.02	0.01	4.72	0.05	0.34
**Mo**	0.05	0.05	0.05	0.05	0.05	0.56	0.05	0.09	0.04	0.35	0.05	0.06
**Cd**	0.11	0.18	0.14	0.14	0.01	0.07	0.01	0.02	0.00	2.06	0.01	0.12
**Ba**	0.78	12.54	2.20	2.75	0.42	30.83	2.63	4.48	0.98	49.35	3.23	4.48
**W**	0.05	0.05	0.05	0.05	0.05	4.48	0.05	0.15	0.05	0.05	0.05	0.05
**Pb**	0.05	3.77	0.27	0.51	0.05	16.33	0.05	0.54	0.13	13.36	0.45	1.17

**Table 2:** Comparison of Ni and Pb concentrations in snowmelt with EU Environmental Quality Standards (EQSbioavailable).

Element	EQSbioavailable (µg/L)	Range in dataset 2021–2023 (µg/L)
Ni	4.0	0.05 – 14.21
Pb	1.2	0.05 – 16.33

The results presented in Table 2 indicate that nickel and lead concentrations at several urban monitoring sites exceeded the corresponding EU Environmental Quality Standards, suggesting potential ecological risks in the study area.

## 4 Experimental Design, Materials and Methods

### 4.1 Location

**Fig. 1:**
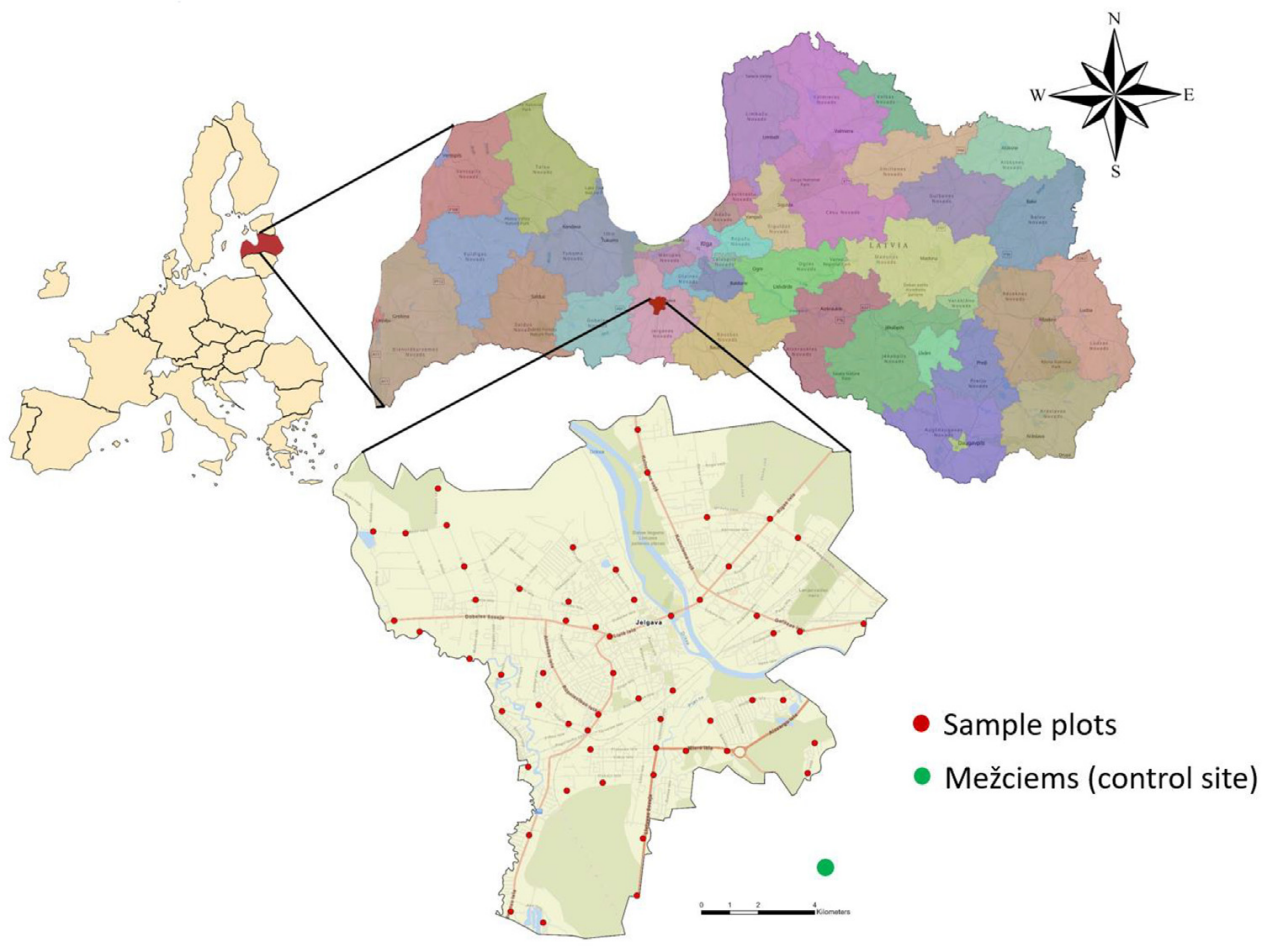
Location of the snow sample collection area, coverage of monitoring points in the Jelgava administrative territory (in red) and location of the rural monitoring point (in green).

Sampling was carried out in Jelgava City (area 60 km²) and at one control site in Mežciems, a rural forest area outside Jelgava with no industrial activity, used as a background reference point (Fig. 1). Within the city boundaries, 59 sampling sites were selected to represent various types of urban land use, including multi-storey residential areas, parks, single-family housing districts, main transport corridors, and industrial zones. The distribution of sampling sites ensured approximately one monitoring point (with three sample collections) per 1 km², following the principles of representative urban monitoring as described in [4,5].

### 4.2 Snow sampling

Snow samples were collected annually in the winters of 2021, 2022, and 2023 during the first snow event after at least six day deposition period, using methods adapted from [6,7]. At each site, three replicates were taken 5 m from the road edge with a 25 cm diameter Teflon-coated steel ring. Snow depth ranged from approximately 5 to 12 cm. Samples were placed in clean polyethylene containers, sealed, and transported to the laboratory.

### 4.3 Sample preparation and physicochemical analysis

In the laboratory, snow samples were melted at room temperature and acidified to 1 % HNO₃ (TraceMetalGrade, 69 %). After 72 h, samples were filtered through pre-washed (1 % HNO₃) ashless paper filters (Whatman 541). Concentrations of Al, Si, Cr, Mn, Fe, Ni, Cu, Zn, As, Mo, Cd, Ba, W, and Pb were determined using inductively coupled plasma mass spectrometry (ICP-MS, Agilent 8900 ICP-QQQ) equipped with a MicroMist nebulizer and helium collision/reaction cell, following established protocols for trace element determination [[8], [9], [10]].

Calibration was performed using certified multi-element standard solutions (10 mg/L, High Purity Standards, ICP-MS-68A, NIST SRM 3100) with external calibration and blank correction across a range of 0.1–100 µg/L. An internal standard mix (Bi, Ge, In, *Sc*, Tb, Y, Li; 10 µg/L) was used to monitor stability. Instrument parameters were: RF power 1550 W, sampling depth 8 mm, auxiliary gas flow 0.90 L/min, plasma gas flow 15 L/min, helium gas flow 5 mL/min.

### 4.4 Quality control

A calibration verification standard was analysed after every 10 samples. Field blanks, laboratory blanks, and spiked samples were included to assess contamination and recovery rates, in line with quality assurance recommendations for ICP-MS trace metal analysis [8,10]. The accuracy of the analytical method was verified through spike recovery tests, since certified reference materials (CRMs) were not available for this study. Spiked snow samples were analysed in parallel with the regular samples to assess the recovery of each element. The obtained recoveries ranged between 92 % and 107 %, which is within the generally accepted range for ICP-MS trace element analysis in environmental samples [8,10].

*The relative standard deviation (RSD) of replicate analyses was below 3–4* *%, depending on the concentration level.*

To ensure analytical transparency, method detection limits (LoD) and limits of quantification (LoQ) were estimated from blank sample measurements using the 3σ and 10σ criteria, respectively. Results reported as “<” indicate concentrations below the method detection limit (LoD). All results below the LoD are consistently reported as “<LoD” in the dataset to ensure transparency and comparability across sampling years. These values represent typical analytical sensitivity under the conditions of the present study and may vary slightly between measurement series due to instrument sensitivity, operating parameters, and sample matrix effects.

The obtained LoD and LoQ values for all analysed elements are presented in Table 3.

**Table 3:** Method detection limits (LoD) and limits of quantification (LoQ) for analysed elements, estimated from blank sample measurements (µg/L)*.*

Element	LoD (µg/L)	LoQ (µg/L)
Al	1.08	3.60
Si	1.00	3.33
Cr	0.02	0.07
Mn	0.10	0.33
Fe	1.00	3.33
Ni	0.06	0.20
Cu	0.07	0.23
Zn	0.20	0.67
As	0.01	0.03
Mo	0.05	0.17
Cd	0.01	0.03
Ba	0.02	0.07
W	0.05	0.17
Pb	0.02	0.07

### 4.5 Meteorological data

Meteorological conditions during the snow deposition period—daily air temperature, precipitation, wind speed, and wind direction—were obtained from the Latvian Environment, Geology and Meteorology Centre [11], following the approach used in Grinfelde et al. [7] for contextualising pollutant deposition.

**Fig. 2:**
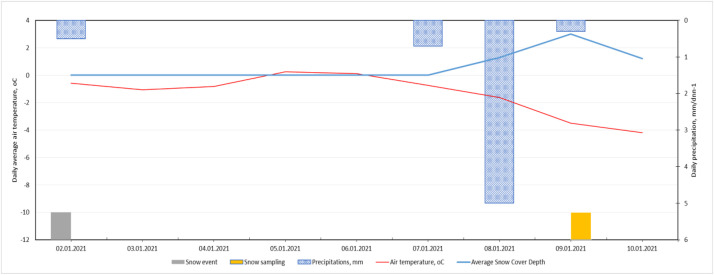
Weather conditions from the first snowfall to sample collection in 2021.

**Fig. 3:**
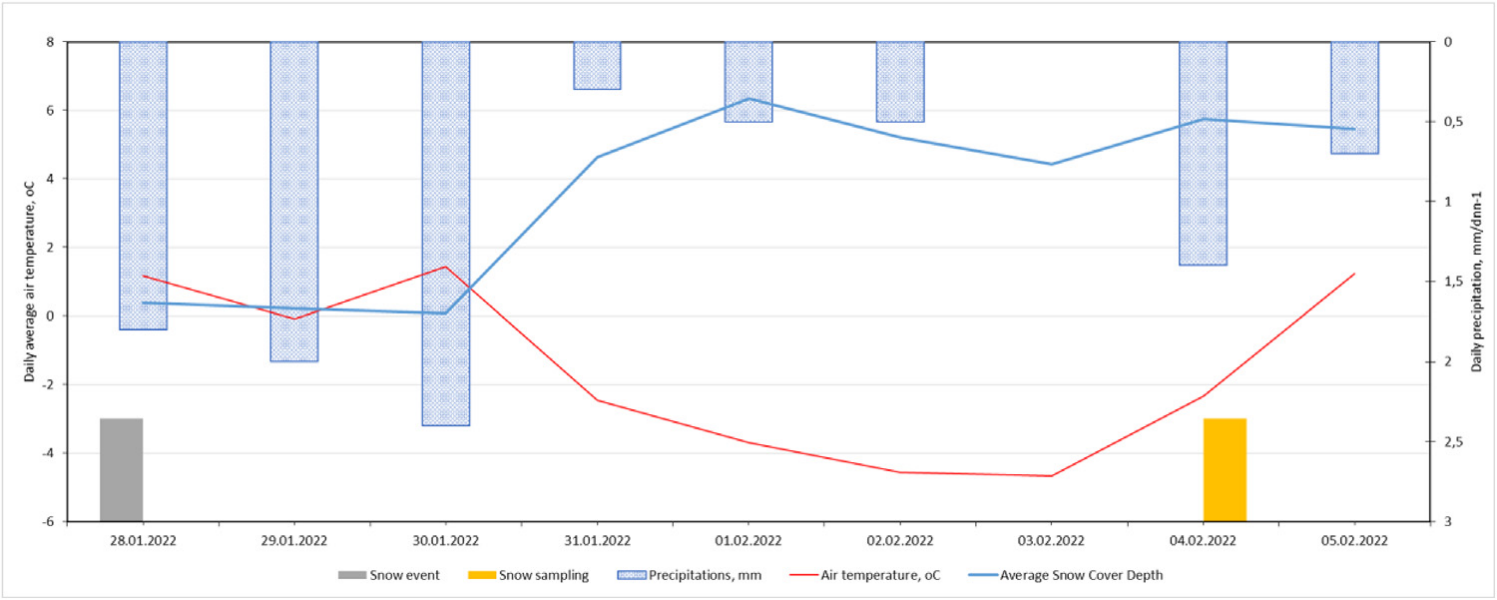
Weather conditions from the first snowfall to sample collection in 2022.

**Fig. 4:**
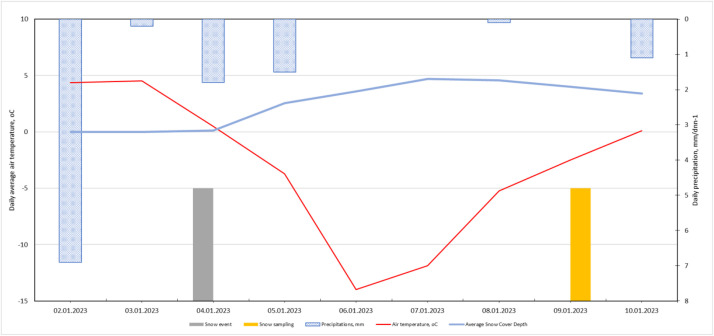
Weather conditions from the first snowfall to sample collection in 2023.

Average daily temperature, precipitation, and snow cover thickness are shown in Figs. 2, 3, and 4. The images show the first snowfall (grey bar) and the day of snow removal (yellow bar).

## Limitations

This dataset has several limitations that should be considered when interpreting the results. First, the observations cover only three years (2021–2023), which restricts the possibility of identifying long-term trends. Second, sampling was carried out once per winter, without seasonal coverage. Third, the results are influenced by meteorological conditions, including variations in snow cover thickness and precipitation during the deposition period. Finally, there is a potential risk of secondary contamination during snow melting prior to analysis. Despite these constraints, the dataset provides a valuable snapshot of urban atmospheric deposition in Jelgava.

## Ethics Statement

Authors confirm that they have read and have followed the ethical requirements for publication in Data in Brief and confirm that the current work does not involve human subjects, animal experiments, or any data collected from social media platforms.

## CRediT Author Statement

**Jovita Pilecka-Ulcugaceva:** Writing-Original Draft, Investigation, Methodology, Data Curation, Writing-Reviewing and Editing. **Inga Grinfelde:** Supervision, Conceptualization, Methodology, Validation. Investigation, **Anda Bakute:** Writing - Review & Editing. **Maris Bertins:** Formal analysis, Writing-Reviewing and Editing. **Arturs Viksna:** Methodology, Resources. **Sindija Frienberga:** Visualisation. **Kristaps Siltumens:** Data Curation.

## Data Availability

Mendeley Data2022 snow data in the city of Jelgava (Original data). Mendeley Data2023 snow data in the city of Jelgava (Original data). Mendeley Data2021 snow data in the city of Jelgava (Original data).
